# Global terrestrial distribution of N_2_O-reducing *Acidobacteriota* members

**DOI:** 10.1093/ismejo/wrag073

**Published:** 2026-03-27

**Authors:** Kazumori Mise, Sawa Wasai-Hara, Hideomi Itoh

**Affiliations:** Biomanufacturing Process Research Center, National Institute of Advanced Industrial Science and Technology (AIST) Hokkaido, 2-17-2-1 Tsukisamu-higashi, Toyohira, Sapporo, Hokkaido 062-8517, Japan; Environmental Biology Division, Institute of Low Temperature Science, Hokkaido University, Kita 19, Nishi 8, Kita-ku, Sapporo, Hokkaido 060-0819, Japan; Biomanufacturing Process Research Center, National Institute of Advanced Industrial Science and Technology (AIST) Hokkaido, 2-17-2-1 Tsukisamu-higashi, Toyohira, Sapporo, Hokkaido 062-8517, Japan; Biomanufacturing Process Research Center, National Institute of Advanced Industrial Science and Technology (AIST) Hokkaido, 2-17-2-1 Tsukisamu-higashi, Toyohira, Sapporo, Hokkaido 062-8517, Japan

**Keywords:** *Acidobacteriota, nosZ*, nitrous oxide, soil, metagenome

## Abstract

Nitrous oxide (N_2_O) is a potent greenhouse gas, and soil is its largest terrestrial source. Microbial N_2_O reductase (NosZ) is the only known enzyme capable of reducing N_2_O to N_2_, making *nosZ*-harboring prokaryotes important sinks in terrestrial ecosystems. Despite being among the most abundant and ubiquitous bacterial phyla in soil, the potential role of *Acidobacteriota* in N_2_O reduction remains largely unexplored. In this study, we addressed this gap using genomic, metagenomic, and physiological analyses. We first analyzed 199,602  prokaryotic genomes, including genomes from both isolated strains and metagenome-assembled genomes (MAGs). We found that 491 *Acidobacteriota* genomes harbored *nosZ*, predominantly the Sec-dependent NosZ gene (*nosZ*II). Global metagenomic analysis of 321 soil samples revealed that *Acidobacteriota nosZ*II is one of the most abundant groups of *nosZ* and distributed across different continents. Among *Acidobacteriota*, *nosZ*II from the class *Vicinamibacteria* was the most prevalent in the soils. Finally, we provide the physiological evidence of N_2_O-reducing activity in *Acidobacteriota* by demonstrating that the *Vicinamibacteria* type strain, *Luteitalea pratensis* KCTC52215^T^, can reduce N_2_O. Taken together, these findings highlight the previously overlooked potential role of *Acidobacteriota* as a global N_2_O sink and underscore the need to include them in future studies on soil N_2_O dynamics.

## Main text

In this era of global climate change, microbial communities that mitigate greenhouse gases have attracted increasing attention. Nitrous oxide (N_2_O) is a potent greenhouse gas with a global warming potential 265 times higher than that of CO_2_, and soil is recognized as a major emission source [1]. Prokaryotes harboring N_2_O reductase (NosZ) serve as crucial N_2_O sinks, and their diversity and distribution have been extensively investigated [2, 3]. NosZI (also known as clade I, typical, or TAT-dependent NosZ) of *Pseudomonadota* and NosZII (clade II, atypical, or Sec-dependent NosZ) of broader prokaryotic phyla, including *Myxococcota*, *Verrucomicrobiota*, and *Gemmatimonadota*, are considered dominant in soils [4, 5]. Prevalence of NosZIII (clade III, lactonase-type, or L-NosZ), a recently reported group of NosZ, has also been argued [6].

Although *Acidobacteriota* (formerly *Acidobacteria*) is one of the most abundant bacterial phyla in soils [7], its potential role in N_2_O reduction remains largely unexplored. A recent study has identified *nosZ* genes in several metagenome-assembled genomes (MAGs) of *Acidobacteriota* [8]. Another work indicated the dominance of acidobacterial *nosZ* at a tundra site [9]. Furthermore, three previously isolated strains (Table S1) harbor *nosZ* homologs; however, their N_2_O-reducing activities have not been demonstrated. These observations suggest the overlooked involvement of *Acidobacteriota* in N_2_O reduction. We aimed to evaluate the importance of *Acidobacteriota* in soil N_2_O reduction through a meta-analysis of global metagenomic data and N_2_O-reducing activity assay in a *nosZ*-harboring type strain.

We first analyzed 199,602 bacterial and archaeal genomes, including MAGs [10–12], 10,909  of which were members of *Acidobacteriota*, to elucidate the distribution of *nosZ* homologs (see Supplementary File for detailed methods). The taxonomic names described below are based on GTDB R226 [12] or Greengenes2 [13], which emphasizes consistency with GTDB. We found that 491 of the *Acidobacteriota* genomes harbored one or more copies of *nosZ* homologs, of which 481 (98.0%) harbored *nosZ*II. Although the acidobacterial NosZ sequences were not monophyletic among those of other prokaryotes, we observed several distinct groups of NosZ that were largely composed of *Acidobacteriota* (outer strip of Fig. S2). In contrast, *nosZ*III was found on none of the *Acidobacteriota* genomes, although we used a low bitscore threshold to minimize false negatives (Fig. S3).

**Figure 1:**
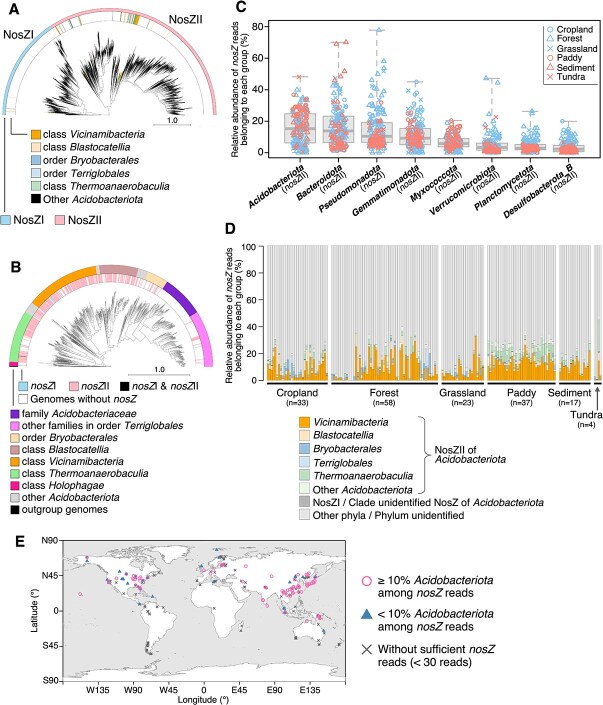
Diversity and prevalence of *nosZ* encoded on *Acidobacteriota* genomes. (A) A phylogenetic tree of NosZ sequences detected on prokaryotic genomes. The outer arc indicates two distinct clades of NosZ, NosZI and NosZII. *Acidobacteriota* NosZ are indicated by colors in the inner arc. (B) A phylogenetic tree of *Acidobacteriota* genomes, comprising of *nosZ*-harboring genomes and near-complete genomes that lack *nosZ*. The outer arc indicates family/order/class names of the genomes. The inner arc represents presence/absence and clade of *nosZ* on each genome. (C) Phylogenetic composition of *nosZ* in shotgun metagenomic datasets from soils. Only datasets with ≥30 reads of *nosZ* are presented (N = 172). Six different land usages are represented by different symbols and colors. Three quartile values are indicated by solid horizontal lines of the boxplot. Whiskers denote the maximum and minimum values. (D) Breakdown of acidobacterial NosZ composition. (E) A map representing the distribution of *Acidobacteriota nosZ* in metagenomes. Sampling locations of metagenomic datasets used in this study are spotted. Shapes and colors of the symbols represent the dominance of *Acidobacteriota nosZ* and number of *nosZ* reads detected.

The distribution of NosZ among *Acidobacteriota* presented a more complicated pattern (Fig. 1B). The lack of *nosZ* is unlikely to be a byproduct of genome incompleteness, as Fig. 1B includes only genomes harboring *nosZ* or a completeness of >99%. There was a complete lack of *nosZ* sequences in all 147 genomes of the family *Acidobacteriaceae* (formerly subdivision 1 member [14]). This absence may be attributed to contrasting physiological traits of this family and the NosZ enzyme. Some NosZ is susceptible to low pH [15, 16], but known *Acidobacteriaceae* isolates are generally acidophilic [17]. In addition, many known isolates of *Acidobacteriaceae* are aerobic [18], whereas N_2_O reduction is prominent in anoxic environments.

We investigated the distribution of *nosZ* genes among microbial communities. The amount of false positive hits for *nosZ* was likely negligible (see Supplementary Note). Here, we discuss the results from 172 of the 321 samples that contained 30 or more reads of *nosZ*. We found that *Acidobacteriota nosZ*II was the most dominant group in 61 of the 172 samples analyzed, with an average relative abundance of 16.1% (median: 15.3%), which was among the highest clade–phylum pairs (Fig. 1C). Other dominant groups included *nosZ*II of the phyla *Bacteroidota*, *Gemmatimonadota*, and *Myxococcota*, and *nosZ*I of the phylum *Pseudomonadota*, which is consistent with previous reports [4, 5]. Among *Acidobacteriota nosZ*, *nosZ*II from class *Vicinamibacteria* (roughly congruent to subdivision 6 [14]) was the most prevalent and dominant, followed by those of class *Thermoanaerobaculia* (mainly in paddy soils) and order *Bryobacterales* (in some upland samples) (Fig. 1D). Conversely, order *Terriglobales*, which includes family *Acidobacteriaceae*, was hardly represented in *nosZ* composition, as expected from the observation that *Terriglobales* genomes seldom harbor *nosZ* (Fig. 1B). In addition, soils dominated by *Acidobacteriota nosZ* were distributed across multiple continents (Fig. 1E).

Both *Vicinamibacteria* and *Terriglobales* are soil-specific, but their pH preferences contrast with each other: members of *Terriglobales* tend to prefer lower pH, whereas those of *Vicinamibacteria* do not [19, 20]. In fact, the relative abundances of 16S rRNA genes were negatively correlated with each other (n = 321, Spearman’s rho = −0.672; Fig. S1), suggesting that *Vicinamibacteria* and *Terriglobales* tend to occupy contrasting ecological niches. Additionally, the frequency of *nosZ* in the metagenomes was negatively and positively correlated with the relative abundances of *Terriglobales* and *Vicinamibacteria* 16S rRNA genes, respectively (Spearman’s rho = −0.492 and 0.356). This aligns with the acid-susceptible characteristics of some NosZ [15], but other studies indicate that N_2_O reduction does occur in acidic soils [6, 21]. At present, we cannot naively conclude that the dominance of *Vicinamibacteria nosZ* is a corollary of the similar pH optima for NosZ and *Vicinamibacteria*.

Some acidobacterial NosZ are scattered throughout the phylogenetic tree (Fig. 1A). NosZ may be a product of recent horizontal gene transfer, and annotations based on such reference sequences can be unreliable. However, homologs of acidobacterial reference sequences forming major clusters (outer strip in Fig. S2A) occupied a large portion of the reads (Fig. S2B). This indicates that the results of metagenomic annotation were not extensively affected by horizontal gene transfer, although we cannot strictly preclude its influence.

**Figure 2:**
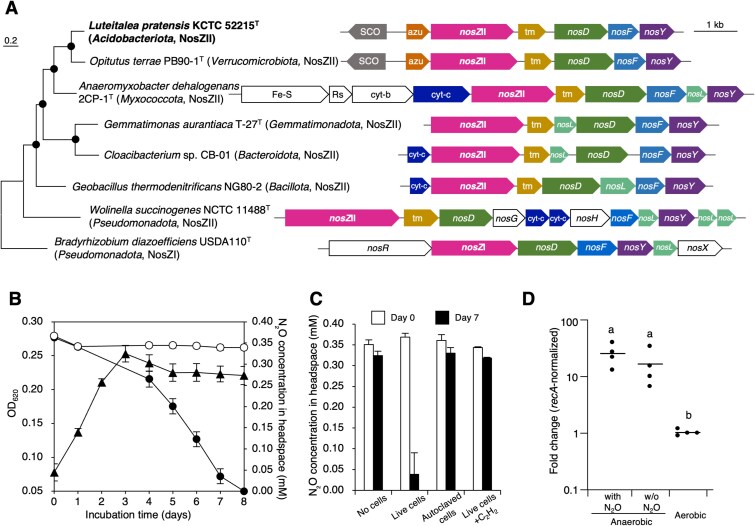
N_2_O-reducing activity of the *Acidobacteriota* strain, *Luteitalea pratensis* KCTC52215^T^, belonging to the class *Vicinamibacteria*. (A) A maximum-likelihood phylogenetic tree based on NosZ amino acid sequences and the genetic organization of the *nos* gene cluster in *L. pratensis* KCTC52215^T^ compared with those of other cultured strains within *nosZ*II-harboring phyla. The NosZI sequences of *Bradyrhizobium diazoefficiens* USDA110^T^ were used as the outgroup. Closed circles indicate bootstrap values >70%. Additional protein labels: SCO, SCO family protein (Cu chaperone); azu, azurin; tm, transmembrane protein; cyt-b, b-type cytochrome; cyt-c, c-type cytochrome; Fe-S, iron–sulfur-binding protein; Rs, Rieske iron–sulfur protein. (B) Time-course changes in headspace N_2_O concentration during cultivation under anoxic conditions with N_2_O. Symbols: ●, N_2_O with cells; ○, N_2_O without cells; ▲, OD_620_. Data represent means ± standard deviations (n = 3). Some error bars are shorter than the symbols. (C) N_2_O reduction by *L. pratensis* KCTC52215^T^ under anoxic conditions. Headspace N_2_O concentrations were measured on Day 0 and Day 7 for cultures containing live cells, autoclaved cells, live cells supplemented with acetylene (C_2_H_2_, an inhibitor of N_2_O reductase), or no cells (medium-only control). Data represent means ± standard deviations (n = 3). (D) Fold changes in *nosZ* transcription levels under different cultivation conditions. Cells were cultivated under anoxic conditions with or without N_2_O addition, or under oxic conditions. Transcription levels of *nosZ* were normalized to those of *recA*, and fold changes were calculated. Each dot represents an individual biological replicate. Horizontal bars indicate mean values (n = 4). Values marked with different letters indicate significant differences (*P* < 0.05).

Based on the metagenomic results, we investigated whether a *nosZ*-harboring *Vicinamibacteria* strain could consume N_2_O. We used the type strain, *Luteitalea pratensis* KCTC52215^T^ [22], which encodes a *nos* gene cluster, including *nosZDFY*, similar to other *nosZ*II-harboring phyla (Fig. 2A), whereas lacking dissimilatory nitrite reductase gene *nirK* or *nirS*. Furthermore, similar to *Opitutus terrae* PB90-1^T^ (*Verrucomicrobiota*), strain KCTC52215^T^ encodes an SCO family protein (a Cu chaperone) and azurin genes upstream of *nosZ* (Fig. 2A), which may facilitate Cu delivery or electron transfer to NosZ. When cultured under strictly anoxic conditions with N_2_O supplied in the headspace (Ar:N_2_O, 99:1, v/v; see Supplementary Methods), strain KCTC52215^T^ consistently decreased the headspace N_2_O concentration in sealed serum vials, indicating active N_2_O reduction (Fig. 2B), whereas such N_2_O reduction observed in live-cell cultures was inhibited in cultures containing autoclaved cells or supplemented with acetylene, an inhibitor of N_2_O reductase (Fig. 2C). *nosZ* transcription levels were significantly higher under anoxic conditions than under oxic conditions, regardless of N_2_O addition (Fig. 2D and S4). N_2_O reduction was primarily observed after cell growth had reached a plateau, suggesting that this activity was not directly coupled to exponential growth (Fig. 2B). These results demonstrate that *L. pratensis* KCTC52215^T^ performs N_2_O reduction, providing the experimental evidence of a functional N_2_O sink within *Acidobacteriota* members.

In conclusion, our genomic, metagenomic, and physiological analyses indicated that members of *Acidobacteriota*, particularly the class *Vicinamibacteria*, are globally prevalent in *nosZ*-associated soil microbiomes and include a lineage that can exhibit N_2_O-reducing activity, highlighting their potential contribution to soil N_2_O elimination. These findings shed new light on the roles of *Acidobacteriota* in N_2_O reduction, which have been overlooked in previous studies, providing a foundation for comprehensive assessments of microbial contributions to soil N_2_O dynamics.

## Supplementary Material

01R2_supplementary_wrag073

## Data Availability

Key intermediate files and bioinformatic scripts are available in FigShare (https://doi.org/10.6084/m9.figshare.30217561).
