# Supplementary material for: Global terrestrial distribution of N2O-reducing Acidobacteriota members
Source: ISME J. 2026 Mar 27;20(1):wrag073. doi: 10.1093/ismejo/wrag073 (PMC13122619; doi:10.1093/ismejo/wrag073)
Supplement: 01R2_supplementary_wrag073 [file 01r2_supplementary_wrag073.pdf]

FULL DETAIL OF METHODS

Exploration of *nosZ*-harboring genomes

To elucidate the diversity and distribution of *nosZ*-harboring prokaryotes, we screened representative genomes of GTDB226 (n = 143,614) [1] and all genomes in the two soil MAG bundles (n = 40,350 and 15,638 for [2] and [3], respectively). We predicted the CDSs in each genome using prodigal version 2.6.3 [4] with the default parameter settings. The predicted amino acid sequences of these CDSs were searched against three HMM profiles of NosZ: TIGR04244.1 (clade I NosZ [NosZI]) and TIGR04246.1 (clade II NosZ [NosZII]) from TIGRFAM [5] and K00376 (covering both types of NosZ) from KOfam version 2025-02-01 [6]. We used HMMER 3.4 [7]. A CDS was regarded as a NosZ if and only if bitscores equal to or above the preset thresholds were obtained for both TIGRFAM (either TIGR04244.1 or TIGR04246.1) and KOfam (K00376). CDSs satisfying the bitscore threshold for both TIGR04244.1 and K00376 were regarded as NosZI, whereas those satisfying the threshold for TIGR04246.1 and K00376 were regarded as NosZII. None of the CDSs satisfied the criteria for both NosZI and NosZII.

Phylogeny of NosZ sequence database

Questionable sequences were discarded from the set of CDS annotated as NosZ. Specifically, we eliminated sequences from genomes with contamination scores (CheckM2) >5% and sequences containing “X” (ambiguous amino acid). The filtered NosZ sequences were aligned using MAFFT v7.525 [8] with the options “--auto --bl 45”, followed by construction of an approximate maximum likelihood tree using FastTree 2.1.11 [9] with default parameter settings. We rerooted the phylogenetic tree at the midpoint of the branch dividing NosZI and NosZII, both of which became monophyletic after rerooting. Here we used the nw_reroot command of Newick Utilities

[10]. Multiple sequence alignment (MSA) of NosZ sequences and their phylogenetic tree were packaged using taxtastic v0.11.1 (distributed along with pplacer).

Phylogeny of *Acidobacteriota* genomes with and without *nosZ*

To examine the phylogenetic distribution of *nosZ* among *Acidobacteriota* genomes, we constructed a phylogenetic tree of *Acidobacteriota* genomes with and without *nosZ*. Here we used *Acidobacteriota* genomes that satisfied one of the following two criteria: (i) genomes harboring *nosZ*; (ii) genomes that did not encode *nosZ* homologs with completeness scores > 99% and contamination scores < 2%. We used completeness or contamination scores calculated using CheckM2 [11]. We also used the following three genomes in GTDB R226 as outgroups: RS_GCF_900111765.1 (*Myxococcus fulvus*), RS_GCF_000196175.1 (*Bdellovibrio bacteriovorus*), and RS_GCF_000022145.1 (*Anaeromyxobacter dehalogenans*).

Target genomes outside the GTDB (i.e., genomes from [2,3]) were fed into GTDB-Tk version 2.4.1 [12]. This generated MSA between their marker genes and those of GTDB genomes. We selected sequences of the target genomes (explained in the previous paragraph) from this MSA, followed by the construction of an approximate maximum likelihood tree using FastTree. The obtained tree was rerooted such that the three genomes mentioned above served as outgroups.

Soil shotgun metagenomic analyses

To investigate the composition of N_2O reducers in soil, we analyzed soil shotgun metagenomic sequences collected worldwide. We reused the quality-filtered nucleotide sequences collected in

[13], which are available at https://doi.org/10.25452/figshare.plus.25332547.v1. The sequences were decompressed using MFCompress [14].

The overall strategies for metagenomic annotations were similar to those described previously [13,15]. We searched for reads encoding *nosZ* or ribosomal protein genes (listed in Table S2). Ribosomal protein genes were used to normalize the number of reads between samples because each of them exists in one copy per prokaryotic cell. To improve annotation efficiency, we performed a two-step homology search. First, we prepared small databases consisting of only NosZ and ribosomal proteins from eggNOG version 5.0.2 [16]. Prior to this, eggNOG database had been screened for NosZ and ribosomal protein sequences. NosZ sequences were identified in the same procedure as described above, and ribosomal proteins were identified using KofamScan version 1.3.0 and KOfam version 2025-02-01 [6,17] with default parameter settings. We mapped all metagenomic reads onto the small databases using the blastx command implemented in DIAMOND v2.1.11 [18], with a "--sensitive" option and an e-value threshold of 1e–5. The mapped reads were regarded as candidates encoding NosZ or ribosomal proteins. Then we performed a second screening, where the candidate reads were subjected to homology search against the full-size eggNOG database (i.e., second screening). Here we identified CDS(s) on each read using prodigal (default settings under "-p meta" flag) and removed CDSs shorter than 50 aa (including the asterisk denoting stop codon). Then we annotated the CDSs using blastp command in DIAMOND with the options "--sensitive -e 1e-5 -k 200". Only the top hits were used to determine the annotation of each query sequence. In cases where multiple CDSs were predicted on one read, the annotation of only one CDS that received the highest bitscore was retained.

To estimate the dominance of *nosZ*-harboring microbes in each metagenome, we calculated the relative abundance of *nosZ* reads to ribosomal protein gene reads. Differences in the lengths of the orthologs were corrected using reads per kilobase of reference sequence per million sample reads (RPKM). Regarding ribosomal protein genes, the gene length used for the RPKM calculation was derived from a previous report (Table S3 in [13]). The length of *nosZ* for RPKM calculation was determined based on the seed sequences of NosZ in TIGRFAM. More specifically, we first calculated the median length of all seed sequences for TIGR04244.1 and TIGR04246.1 (families representing NosZI and NosZII, respectively). We assumed that their median length represents an ordinary length of NosZ. We then tripled the NosZ length to obtain *nosZ* length. The ratio (RPKM of *nosZ*)/(median of RPKMs of ribosomal protein genes) was calculated and used as a proxy for the relative abundance of *nosZ*-harboring microbes.

The NosZ amino acid sequences (encoded on metagenomic reads) were mapped onto the MSA of NosZ sequences (as explained above) using MAFFT v7.525 with the options "--auto --add --bl 45". We then performed phylogenetic placement of NosZ reads using pplacer v1.1.alpha19-0-g807f6f3 with the options "--mrca-class --discard-nonoverlapped". We determined their taxonomy and NosZ clade using rppr v1.1.alpha19-0-g807f6f3 and guppy v1.1.alpha19-0-g807f6f3 with the options "prep_db" and "classify --mrca-class" [19]. The confidence threshold was set at 0.49999 and 0.79999 for taxonomic affiliation and the NosZ clade, respectively, considering the effect of floating-point errors.

To further validate the annotation of acidobacterial NosZ on metagenomic reads, we mapped all *Acidobacteriota* NosZ reads to the NosZ database using Needleman-Wunsch algorithm. We used SWORD v1.0.4 [20] for this mapping with the options "-a 10000 -A HW -v 1e-5". We selected the top hit for each *nosZ* read. The proportion of reads assigned to the major

cluster of acidobacterial NosZ (represented in red in Figure S2) was calculated for each metagenomic sample.

We also analyzed 16S rRNA gene sequences contained in the shotgun metagenomic sequences. We used intermediate files consisting of 16S rRNA gene reads from the global soil metagenomic sequences [13]. Each of these reads was taxonomically annotated using Greengenes2 version 2024.09 [21] and SINTAX implemented in USEARCH version 12.0 [22], with a confidence threshold of 0.5.

Evaluation of false positives in *nosZ* detection from shotgun metagenomic sequences

The C-terminal of NosZ consists of cupredoxin-like domain, which is broadly conserved in diverse organisms and proteins. We tested if short metagenomic reads from non-NosZ cupredoxin genes can be mistakenly annotated as *nosZ* in our bioinformatic methods.

We comprehensively obtained eukaryotic, bacterial, and archaeal cupredoxin proteins (n = 297,810) from InterPro [23] using IPR008972 as the key. The fetched sequences underwent a series of dereplication and filtering as detailed below. First, the sequences were clustered with a similarity threshold of 90% using CD-HIT V4.8.1. Second, we eliminated sequences shorter than 50 aa and those labeled as NosZ or nitrous oxide reductase. Third, we scanned all the remaining sequences using HMMER and profile HMMs of NosZ in the same procedure as we did for prokaryotic genomes. We eliminated sequences that yielded a bitscore above threshold for TIGRFAM or KOfam. From the remaining sequences, we extracted cupredoxin domains (designated as such by InterPro) and flanking 10 aa of both ends. We clustered the extracted subsequences at 90% similarity threshold using CD-HIT and eliminated sequences shorter than 50 aa. From each of the filtered sequences (n = 151,307), we obtained 1–5 randomly selected

subsequences of 50 aa long (n = 754,680). These random subsequences, the mimicry of short-read shotgun sequences, were queried against eggNOG database using DIAMOND in the same parameter settings as the second screening of shotgun metagenomic analysis.

Phylogeny of NosZ and genomic context of *nos* gene clusters among cultured strains

NosZII amino acid sequences from *Luteitalea pratensis* KCTC52215^T and other representative cultured strains within *nosZ*II-harboring phyla were aligned using MAFFT [8] via the EMBL-EBI web server (https://www.ebi.ac.uk/jdispatcher/msa/mafft). A maximum-likelihood phylogenetic tree was constructed using MEGA v12.0.14 [24] where the best-fit substitution model (Le and Gascuel [LG] +Freq; [25]) was selected and branch support was assessed using the adaptive bootstrapping function. The NosZI sequence from *Bradyrhizobium diazoefficiens* USDA110^T was used as an outgroup. The genetic organization of the *nos* gene clusters in *L. pratensis* KCTC52215^T and other cultured strains was manually examined for CDSs near NosZ; transmembrane proteins were predicted using DeepTMHMM (https://services.healthtech.dtu.dk/services/DeepTMHMM-1.0 [26]), and the functions of other proteins were annotated using InterProScan (https://www.ebi.ac.uk/interpro [27]), Conserved Domain (CD) Search (https://www.ncbi.nlm.nih.gov/Structure/cdd/wrpsb.cgi [28]), and KEGG BLASTP (https://www.genome.jp/tools/blast [17]), with reference to a previous report [29]. Gene cluster diagrams were generated using the drawGeneArrows3 tool (Tohoku University, https://www.ige.tohoku.ac.jp/joho/labhome/tool.html, accessed September 13, 2025).

Searching for clade III *nosZ* encoded on *Acidobacteriota* genomes

Clade III *nosZ* (*nosZ*III), which encodes lactonase-like clade III NosZ (NosZIII, L-NosZ), is a distinct group of *nosZ* that was recently reported [30]. To investigate if *Acidobacteriota* own *nosZ*III on their genomes, we constructed an HMM profile that specifically detects NosZIII and used it to search *Acidobacteriota* genomes for *nosZ*III.

We first obtained a set of 259 NosZ sequences, 164 and 95 of which were from "conventional" NosZ (NosZI or NosZII) and NosZIII, respectively [30]. The overall strategy stated below is conceptually related to the determination of adaptive score thresholds in KofamKOALA [6]. We randomly split the 95 NosZIII sequences into two groups: one (n = 43) was for building an HMM profile, and the other (n= 52) was used for positive validation of the HMM profile. Here we used SeqKit v2.10.0 with options "sample -s 11 -p 0.5". The former set of sequences were aligned using MAFFT (default parameters under "--auto" mode) and an HMM profile was built using hmmbuild command (default parameters under "--amino" mode) implemented in HMMER. We then tested if the HMM profile accurately distinguishes NosZIII from conventional NosZ (i.e., NosZI and NosZII). We used 52 NosZIII sequences and 164 NosZI/II sequences (described above) as positive and negative training data, respectively. These sequences were aligned with the HMM profile using "hmmscan" command of HMMER. Based on the distributions of bitscores of the positive and negative validation datasets (Figure S3), we determined that any bitscore threshold between 300–700 (full-length score) or 250–700 (best domain score) would yield a perfect distinction between NosZIII and NosZI/NosZII.

Using the HMM profile of NosZIII, we searched for NosZIII from all CDS amino acid sequences of genomes used in this study. We used "hmmscan" command implemented in HMMER with default parameter settings. After the HMM search, we filtered hits with full-length

bitscores of ≥ 300 or best-domain bitscores of ≥ 250. These thresholds were used to minimize false negatives within the ranges mentioned above.

Throughout the bioinformatic analyses, we used SeqKit v2.10.0 [31], taxonkit v0.20.0 [32], and iTOL [33] to format fasta/fastq files, to manage taxonomy names, and to visualize phylogenetic trees, respectively. R version 4.4.2 [34] was also used for visualization and statistical analyses.

Measurement of N_2O-reducing activities of a *nosZ*-harboring acidobacterial strain

The type strain *L. pratensis* KCTC 52215^T [35] belonging to the class *Vicinamibacteria* was obtained from the Korean Collection for Type Cultures (KCTC). Cultivation was performed in a modified SSE/HD 1:10 medium (DSMZ Medium 1426; hereafter referred to as mSSE), in which MES was replaced with HEPES (pH 7.0), and the compositions and concentrations of the vitamin and trace element solutions were slightly altered (complete formulation provided in Tables S3 and S4). Prior to inoculation, the medium was thoroughly degassed by flushing with Ar to remove dissolved oxygen. The trace element and vitamin solutions were added at final concentrations of 10 mL L^{-1} and 5 mL L^{-1}, respectively. For N_2O reduction assays, 16 mL gas-tight serum vials were filled with 3 mL of mSSE medium, sealed with butyl rubber stoppers and aluminum crimp seals, and the headspace was replaced with Ar:N_2O (99:1, v/v) after inoculation with strain KCTC 52215^T. Cultures were incubated at 25 °C with shaking at 150 rpm. Headspace N_2O concentrations were quantified using a gas chromatograph (GC3210, GL Sciences Inc., Tokyo, Japan) equipped with a thermal conductivity detector and a Shincarbon-ST 50/80 column (Shinwa Chemical Industries Ltd., Kyoto, Japan), operated with helium as the carrier gas at a flow rate of 35 mL min^{-1}, a column oven temperature of 150 °C, and injection and detector

temperatures of 210 °C. Calibration curves were generated using standard N_2O gas at concentrations of 2, 1, 0.2, 0.04, and 0.02%. Cell growth was monitored by measuring optical density at 620 nm (OD_{620}) using a spectrophotometer (Miniphoto 620, Taitec, Saitama, Japan). Each condition was tested using three biological replicates (n = 3), and the uninoculated medium served as a negative control for N_2O reduction. In addition, N_2O reduction was compared among cultures containing live cells, autoclaved cells, and live cells supplemented with 10% acetylene (C_2H_2, an inhibitor of N_2O reductase [36]), as well as uninoculated medium. Headspace N_2O concentrations were measured on Day 0 and Day 7 to assess the N_2O-reducing activity under each condition.

To evaluate the transcriptional activity of *nosZ*, cells were cultivated under anoxic or oxic conditions. For cultivation under anoxic conditions, the vial headspace was filled with either 100% Ar (without N_2O) or an Ar:N_2O mixture (99:1, v/v). For cultivation under oxic conditions, the headspace was filled with air. After five days of cultivation, RNA was extracted using the NucleoSpin RNA Plus kit (Macherey-Nagel, Düren, Germany), followed by DNase treatment with the TURBO DNA-free Kit (Thermo Fisher Scientific, MA, USA). Complementary DNA was synthesized with ReverTra Ace qPCR RT Master Mix containing gDNA Remover (Takara Bio, Shiga, Japan), using equal amounts of total RNA from each replicate (n = 4). Quantitative PCR (qPCR) was performed using THUNDERBIRD Next SYBR qPCR Mix (Toyobo, Osaka, Japan) on a LightCycler 96 system (Roche, Basel, Switzerland) with primer sets specific for *nosZ*, *recA*, *rpoB*, or *sigA* (Table S5). Transcription levels of *nosZ* were normalized to each housekeeping gene, and fold changes were calculated. Thermal cycling conditions were as follows: initial denaturation at 95 °C for 30 s, followed by 40 cycles of 95 °C for 5 s and 60 °C for 30 s. Statistical significance among treatments was evaluated on log2-transformed fold

changes using a linear model followed by Tukey's HSD test for all pairwise comparisons (α = 0.05), implemented in R version 4.4.2 [34]. Log2 transformation was performed to approximate normality, and the normality of each treatment group was confirmed using the Shapiro–Wilk test (all groups $P > 0.05$).

SUPPLEMENTARY NOTE

Here we discuss the possible false positives in *nosZ* detection from shotgun metagenomic sequences. NosZ is a member of cupredoxin protein, and its C-terminal domain is somewhat homologous to other cupredoxins. This may indicate that metagenomic reads encoding cupredoxin genes other than *nosZ* may be mistakenly determined as *nosZ*.

We generated 754,680 subsequences of 50 aa length from non-NosZ cupredoxin sequences in InterPro. The sequences were mapped onto eggNOG database using DIAMOND in the same condition as we did for shotgun metagenomic analysis. As a result, only 14 sequences (< 0.002%) of the query sequences were annotated as NosZ. The 14 sequences were further fed into the taxonomic annotation pipeline (the same tools and settings as metagenomic analysis), and none of them were determined as *Acidobacteriota* NosZ. The results indicate that our metagenomic analyses and conclusions are largely unaffected by the false detection of non-*nosZ* cupredoxin genes as *nosZ*.

Table S1. A list of known homologs of *nosZ* encoded on pure isolate genomes of *Acidobacteriota*.

Assembly ID	Taxonomy	NosZ ID	Remark	Ref
CP015136.1<br>GCF_001618865.1	*Luteitalea pratensis* DSM 100886^T	WP_110174615.1	*nosZ*II	[35]
AP024452.1<br>GCF_016865485.1	*Luteitalea* sp. strain TBR-22	WP_239492388.1	*nosZ*II	[37]
CP071793.1<br>GCF_017498545.1	*Sulfidibacter corallicola* M133^T	WP_237380149.1	*nosZ*I	[38]

Table S2. A list of marker genes and their lengths used for metagenomic analyses in this study. Provided in a separate Excel spreadsheet.

Table S3. Composition of the vitamin solution (per 1 L). Provided in a separate Excel spreadsheet.

Table S4. Composition of the trace element solution (per 1 L). Provided in a separate Excel spreadsheet.

Table S5. Primer sequences used for quantitative PCR in this study.

Target gene	Primer name	Sequence (5´–3´)
nosZ	nosZ_F	TGTTCTACTCGGGTGGTCAC
	nosZ_R	CTTGCCCATCATCGACTTGG
recA	recA_F	CAAGATCGTCGACAAGAGCG
	recA_R	GTCGCCGGATTGTCTTTCAG
rpoB	rpoB_F	CAAGAACCTGCTGTACGTGC
	rpoB_R	TTGAGGTGCAGGGAATCGAT
sigA	sigA_F	ACACGCTGGAGACGATCTAC
	sigA_R	CGGTTGGTGTACTTCTTGGC

Figure S1. Correlations between the relative abundance of *nosZ* in metagenomes (corrected by RPKMs of ribosomal protein genes) and the relative abundances of 16S rRNA gene reads annotated as class *Vicinamibacteria* or order *Terriglobiales*. Each point denotes one metagenomic sample (N = 321). Results of Spearman's correlation test between two factors are also indicated in each panel.

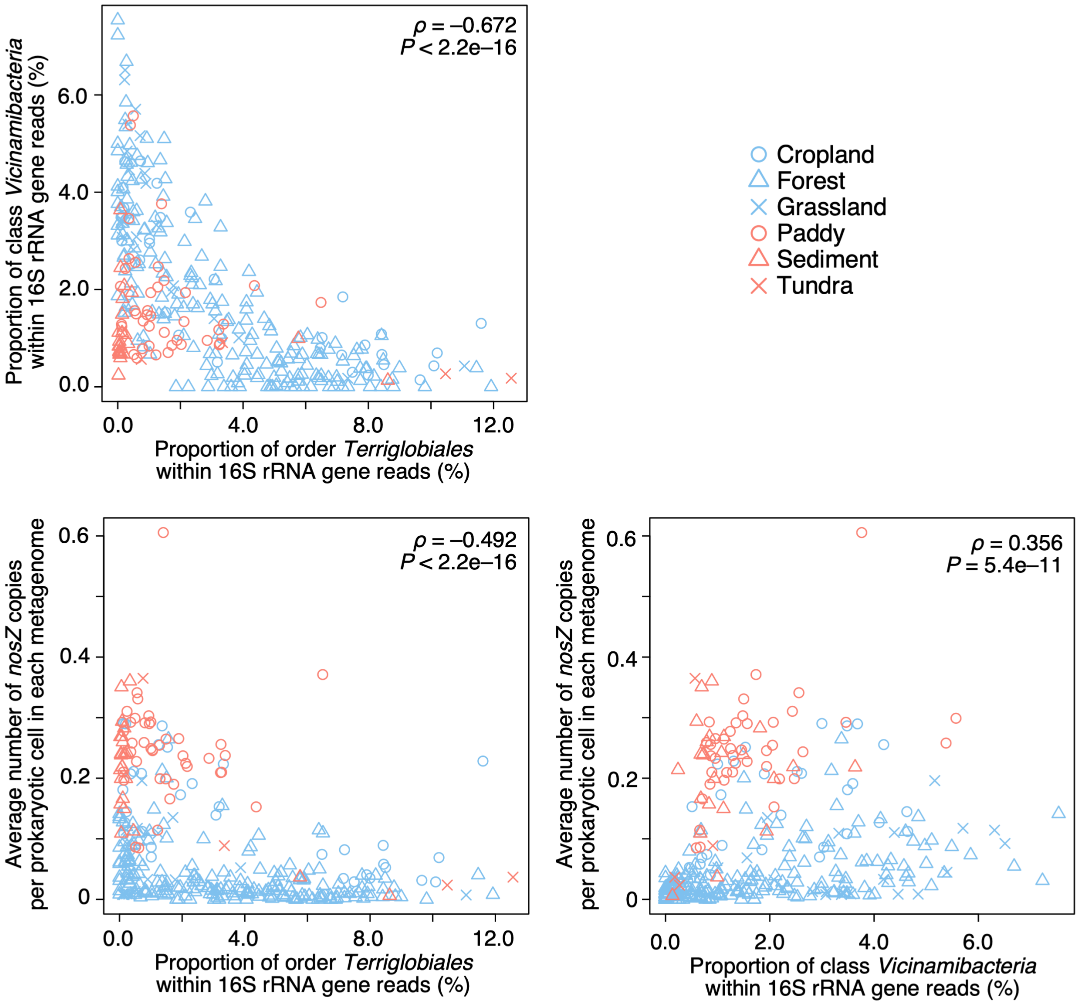

Figure S2. (**A**) A phylogenetic tree of NosZ sequences. Red stripes indicate major clusters of acidobacterial NosZ that are phylogenetically conserved. (**B**) Dominance of acidobacterial *nosZ* reads that were mapped onto the reference sequences included in major clusters of acidobacterial NosZ.

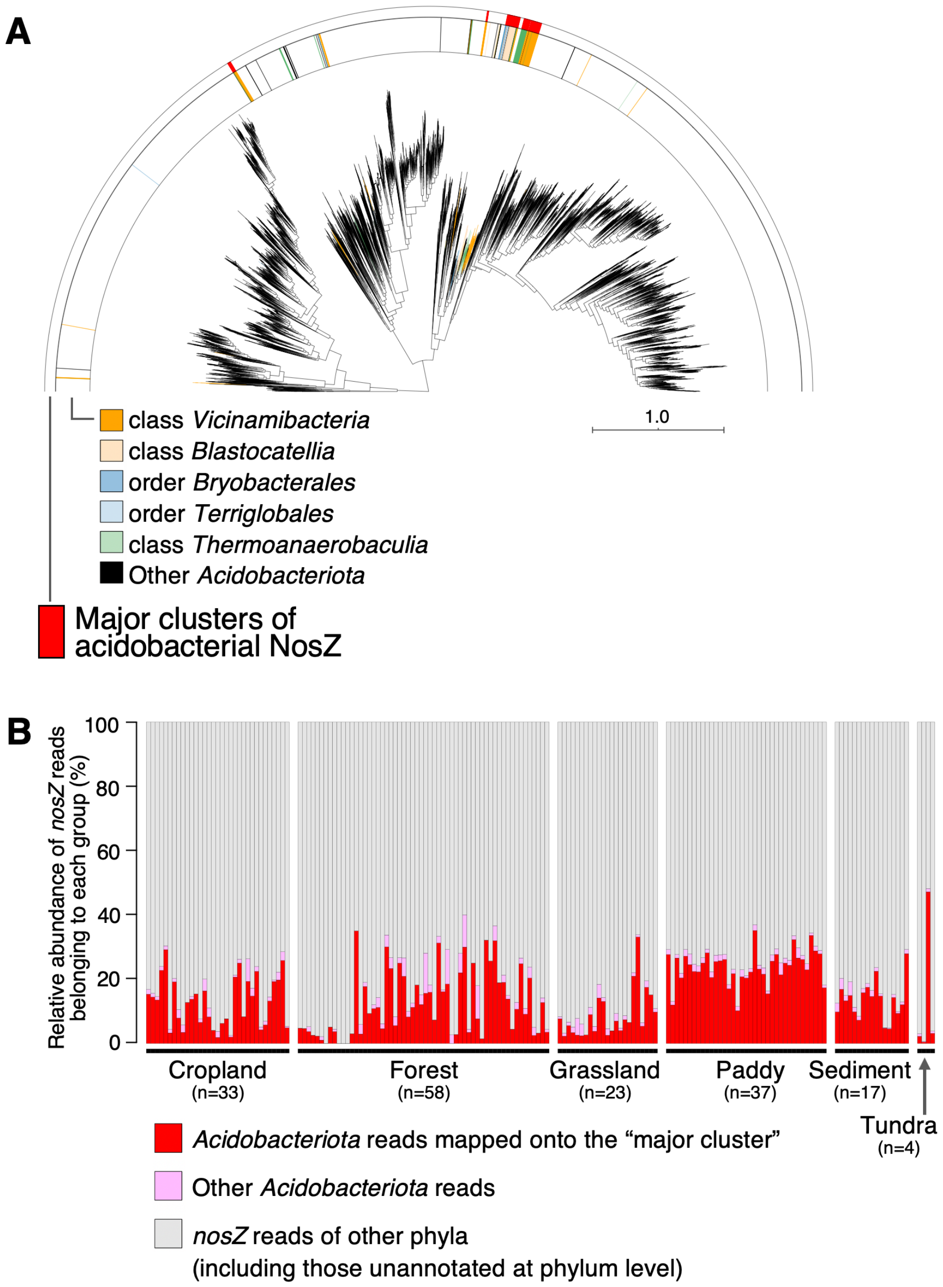

Figure S3. Bitscore distributions of NosZ sequences mapped onto HMM profile of NosZIII. Different colors indicate bitscores of two different datasets, namely positive validation data and negative validation data. (**A**) Distribution of full-length bitscores. (**B**) Distribution of best-domain bitscores.

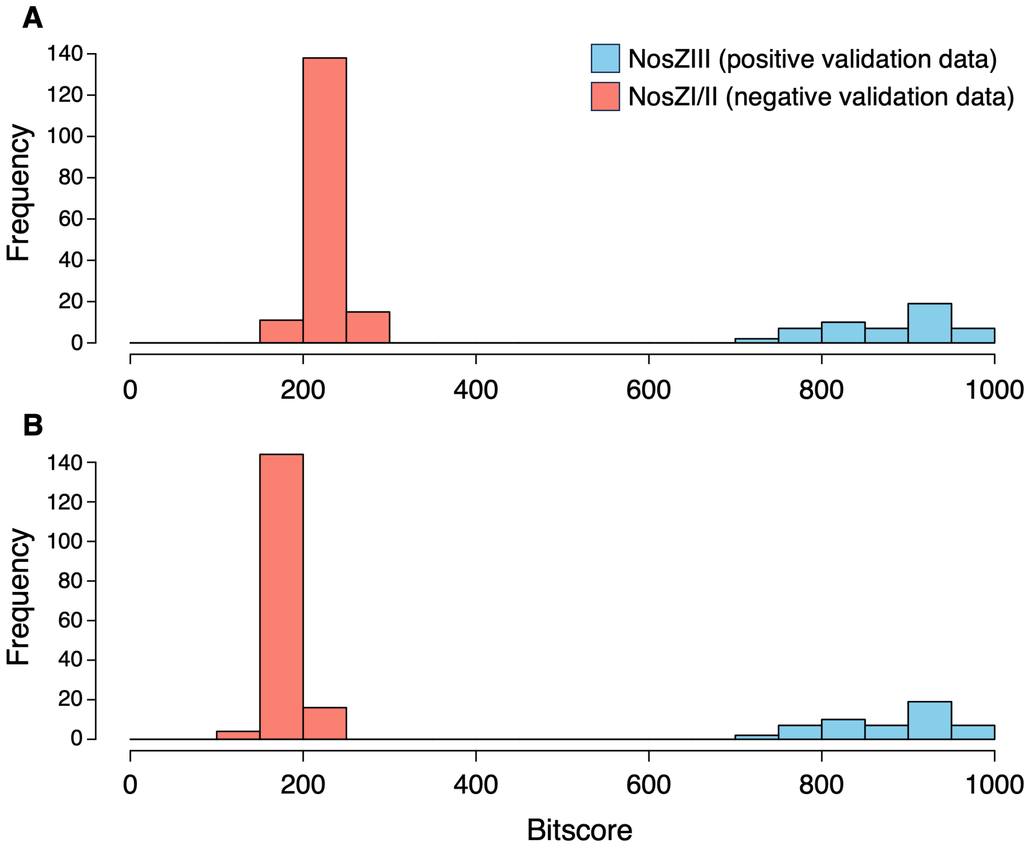

Figure S4. Fold changes in *nosZ* transcription levels under different cultivation conditions. Cells were cultivated under anoxic conditions with or without N_2O addition, or under oxic conditions. Transcription levels of *nosZ* were normalized to those of *rpoB* (**A**) and *sigA* genes (**B**), and fold changes were calculated. Each dot represents an individual biological replicate. Horizontal bars indicate mean values (n = 4). Values marked with different letters indicate significant differences ($P < 0.05$).

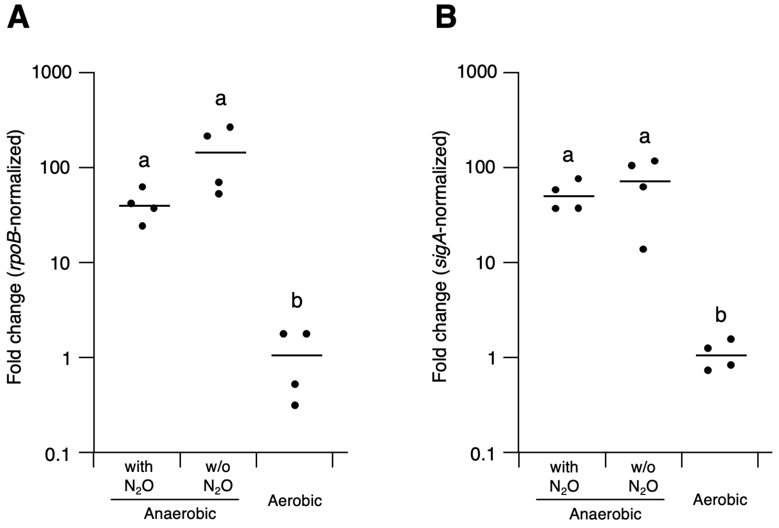

REFERENCES

1. Parks DH, Chuvochina M, Rinke C *et al.* GTDB: an ongoing census of bacterial and archaeal diversity through a phylogenetically consistent, rank normalized and complete genome-based taxonomy. *Nucleic Acids Res* 2022;50:D785–94.
2. Ma B, Lu C, Wang Y *et al.* A genomic catalogue of soil microbiomes boosts mining of biodiversity and genetic resources. *Nat Commun* 2023;14:7318.
3. Sereika M, Mussig AJ, Jiang C *et al.* Genome-resolved long-read sequencing expands known microbial diversity across terrestrial habitats. *Nat Microbiol* 2025;10:2018–30.
4. Hyatt D, Chen G-L, LoCascio PF *et al.* Prodigal: prokaryotic gene recognition and translation initiation site identification. *BMC Bioinformatics* 2010;11:119.
5. Haft DH, Loftus BJ, Richardson DL *et al.* TIGRFAMs: a protein family resource for the functional identification of proteins. *Nucleic Acids Res* 2001;29:41–3.
6. Aramaki T, Blanc-Mathieu R, Endo H *et al.* KofamKOALA: KEGG Ortholog assignment based on profile HMM and adaptive score threshold. *Bioinformatics* 2020;36:2251–2.
7. Eddy SR. Accelerated Profile HMM Searches. *PLoS Comput Biol* 2011;7:e1002195.
8. Katoh K. MAFFT: a novel method for rapid multiple sequence alignment based on fast Fourier transform. *Nucleic Acids Res* 2002;30:3059–66.
9. Price MN, Dehal PS, Arkin AP. FastTree 2 – Approximately Maximum-Likelihood Trees for Large Alignments. *PLoS One* 2010;5:e9490.
10. Junier T, Zdobnov EM. The Newick utilities: high-throughput phylogenetic tree processing in the UNIX shell. *Bioinformatics* 2010;26:1669–70.
11. Chklovski A, Parks DH, Woodcroft BJ *et al.* CheckM2: a rapid, scalable and accurate tool for assessing microbial genome quality using machine learning. *Nat Methods* 2023;20:1203–12.
12. Chaumeil P-A, Mussig AJ, Hugenholtz P *et al.* GTDB-Tk: a toolkit to classify genomes with the Genome Taxonomy Database. *Bioinformatics* 2020;36:1925–7.
13. Masuda Y, Mise K, Xu Z *et al.* Global soil metagenomics reveals distribution and predominance of *Deltaproteobacteria* in nitrogen-fixing microbiome. *Microbiome* 2024;12:95.
14. Pinho AJ, Pratas D. MFCompress: a compression tool for FASTA and multi-FASTA data. *Bioinformatics* 2014;30:117–8.
15. Mise K, Masuda Y, Senoo K *et al.* Betaproteobacterial clade II *nosZ* activated under high N_2O concentrations in paddy soil microcosms. *J Appl Microbiol* 2025;136.

16. Huerta-Cepas J, Szklarczyk D, Heller D *et al.* eggNOG 5.0: a hierarchical, functionally and phylogenetically annotated orthology resource based on 5090 organisms and 2502 viruses. *Nucleic Acids Res* 2019;47:D309–14.

17. Kanehisa M, Furumichi M, Sato Y *et al.* KEGG: biological systems database as a model of the real world. *Nucleic Acids Res* 2025;53:D672–7.

18. Buchfink B, Reuter K, Drost H-G. Sensitive protein alignments at tree-of-life scale using DIAMOND. *Nat Methods* 2021;18:366–8.

19. Matsen FA, Kodner RB, Armbrust EV. pplacer: linear time maximum-likelihood and Bayesian phylogenetic placement of sequences onto a fixed reference tree. *BMC Bioinformatics* 2010;11:538.

20. Vaser R, Pavlović D, Šikić M. SWORD—a highly efficient protein database search. *Bioinformatics* 2016;32:i680–4.

21. McDonald D, Jiang Y, Balaban M *et al.* Greengenes2 unifies microbial data in a single reference tree. *Nat Biotechnol* 2024;42:715–8.

22. Edgar RC. SINTAX: a simple non-Bayesian taxonomy classifier for 16S and ITS sequences. *bioRxiv* 2016, DOI: 10.1101/074161.

23. Blum M, Andreeva A, Florentino LC *et al.* InterPro: the protein sequence classification resource in 2025. *Nucleic Acids Res* 2025;53:D444–56.

24. Kumar S, Stecher G, Suleski M *et al.* MEGA12: Molecular Evolutionary Genetic Analysis Version 12 for Adaptive and Green Computing. *Mol Biol Evol* 2024;41:msae263.

25. Le SQ, Gascuel O. An Improved General Amino Acid Replacement Matrix. *Mol Biol Evol* 2008;25:1307–20.

26. Hallgren J, Tsirigos KD, Pedersen MD *et al.* DeepTMHMM predicts alpha and beta transmembrane proteins using deep neural networks. *bioRxiv* 2022, DOI: 10.1101/2022.04.08.487609.

27. Blum M, Andreeva A, Florentino LC *et al.* InterPro: the protein sequence classification resource in 2025. *Nucleic Acids Res* 2025;53:D444–56.

28. Wang J, Chitsaz F, Derbyshire MK *et al.* The conserved domain database in 2023. *Nucleic Acids Res* 2023;51:D384–8.

29. Hallin S, Philippot L, Löffler FE *et al.* Genomics and Ecology of Novel N_2O-Reducing Microorganisms. *Trends Microbiol* 2018;26:43–55.

30. He G, Wang W, Chen G *et al.* A novel bacterial protein family that catalyses nitrous oxide reduction. *Nature* 2025;646:152–60.

31. Shen W, Le S, Li Y *et al.* SeqKit: A Cross-Platform and Ultrafast Toolkit for FASTA/Q File Manipulation. *PLoS One* 2016;11:e0163962.

32. Shen W, Ren H. TaxonKit: A practical and efficient NCBI taxonomy toolkit. *Journal of Genetics and Genomics* 2021;48:844–50.

33. Letunic I, Bork P. Interactive Tree of Life (iTOL) v6: recent updates to the phylogenetic tree display and annotation tool. *Nucleic Acids Res* 2024;52:W78–82.

34. R Core Team. R: A Language and Environment for Statistical Computing. 2024. R Foundation for Statistical Computing, Vienna, Austria.

35. Vieira S, Luckner M, Wanner G *et al. Luteitalea pratensis* gen. nov., sp. nov. a new member of subdivision 6 *Acidobacteria* isolated from temperate grassland soil. *Int J Syst Evol Microbiol* 2017;67:1408–14.

36. Yoshinari T, Knowles R. Acetylene inhibition of nitrous oxide reduction by denitrifying bacteria. *Biochem Biophys Res Commun* 1976;69:705–10.

37. Yamamoto K, Yoneda Y, Makino A *et al.* Complete Genome Sequence of *Luteitalea* sp. Strain TBR-22. *Microbiol Resour Announc* 2022;11:mra.00455-21.

38. Wang G, Li Y, Liu J *et al.* Comparative Genomics Reveal the Animal-Associated Features of the *Acanthopleuribacteraceae* Bacteria, and Description of *Sulfidibacter corallicola* gen. nov., sp., nov. *Front Microbiol* 2022;13:778535.
